# Re-revision of a patellar tendon rupture in a young professional martial arts athlete

**DOI:** 10.1007/s10195-011-0160-0

**Published:** 2011-10-19

**Authors:** A. Vadalà, R. Iorio, A. M. Bonifazi, G. Bolle, A. Ferretti

**Affiliations:** Orthopaedic Unit and “Kirk Kilgour” Sports Injury Centre, S. Andrea Hospital, University of Rome “Sapienza”, Via Grottarossa 1035, Rome, RM Italy

**Keywords:** Patellar tendon rupture, Re-revision surgery, Rehabilitation protocol

## Abstract

A 27-year-old professional martial arts athlete experienced recurrent right knee patellar tendon rupture on three occasions. He underwent two operations for complete patellar tendon rupture: an end-to-end tenorrhaphy the first time, and revision with a bone-patellar-tendon (BPT) allograft. After the third episode, he was referred to our department, where we performed a surgical reconstruction with the use of hamstring pro-patellar tendon, in a figure-of-eight configuration, followed by a careful rehabilitation protocol. Clinical and radiological follow-ups were realized at 1, 3, and 6 months and 1 and 2 years postop, with an accurate physical examination, the use of recognized international outcome scores, and radiograph and MRI studies. As far as we know, this is the first paper to report a re-revision of a patellar tendon rupture.

## Introduction

Tendon ruptures among young active athletes are common [1, 2]. In particular, ruptures of the patellar tendon occur in patients practicing sports such as soccer, volleyball, basketball, or combative sports like martial arts [3].

There is a lack of papers with a significant number of patients that were surgically treated for this pathology in the literature due to its rarity. Most authors describe case reports of surgical procedures on traumatic, atraumatic, unilateral, or bilateral ruptures. As far as we know, this is the first case report on the outcome of a patient surgically treated for a revision of a revision of a patellar tendon rupture.

## Case report

**Fig. 1:**
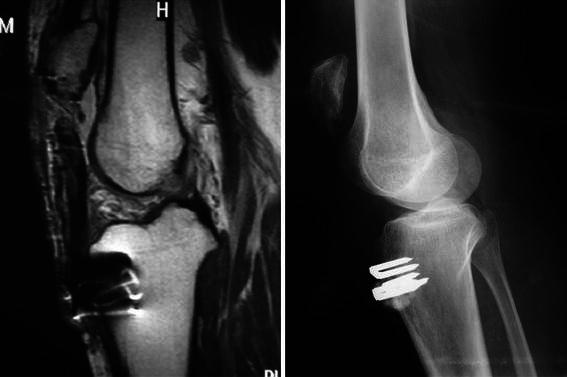
X-ray and MRI images of the torn patellar tendon after the two previous operations

We report the case of a 27-year-old male professional martial arts athlete who experienced a first traumatic patellar tendon rupture in July 2003 during a training session. The diagnosis was initially missed, so it was July 2004 before the patient underwent a surgical operation, where an end-to-end tenorrhaphy was performed. In August 2005 he reported a new re-rupture of the same tendon, and he underwent an immediate surgical revision with a bone-patellar-tendon (BPT) allograft. In January 2007, he experienced a new rupture of the revised tendon and came to our attention; a diagnosis of acute complete re-re-rupture of the right patellar tendon was then made. Upon physical examination, the patient showed a complete lack of active extension, along with a gap at the level of the patellar tendon. Radiograph images showed high patellar bone height; MRI images showed complete rupture of the patellar tendon (Fig. 1).

### Operative technique

**Fig. 2:**
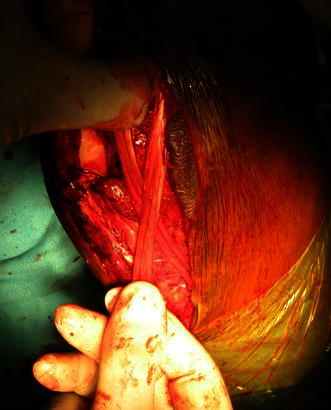
Semitendinosus and gracilis tendons used as augmentation in a figure-of-eight shape. The insertion of the hamstrings on the pes anserinus was left untouched

Autologous gracilis and semitendinosus tendons were harvested from their myotendinous junction, leaving their distal insertion intact: their proximal free ends were prepared with a Bunnel-type suture. After we determined that the hamstrings were long enough to be inserted on the ATT after passage through the patellar tendon in a figure-of-eight shape (Fig. 2), one 6 mm tunnel was made on the patellar bone for the passage of the two tendons. Finally, distal fixation on the ATT was performed once again with a transosseous tunnel. Postoperatively, the knee was immobilized in a full extension brace, and was non-weight bearing for 45 days. He then started progressive partial weight-bearing with the use of crutches. Exercises to regain the range of motion of the knee and to strengthen the quadriceps muscle were started at two months postop. At six months postop, the patient was allowed to run in a straight direction and to strengthen the quadriceps muscle with open kinetic chain exercises.

**Fig. 3:**
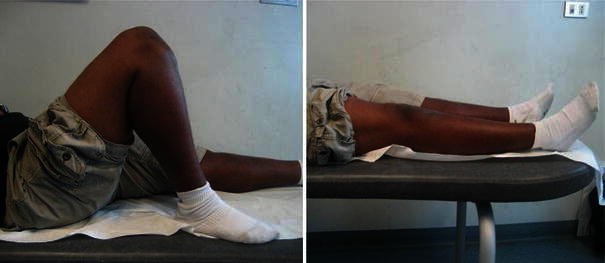
Range of motion at final follow-up

The patient was clinically and radiologically followed up with MRIs at 1, 3, 6, 12, and 24 months after the operation. At final follow-up, the patient reported a satisfactory feeling regarding the surgical procedure performed; the Visual Analog Scale was 2; active range of motion was complete in both flexion and extension (Fig. 3); a 3 cm hypotrophy of the quadriceps muscle was found. The Tegner score was 5 and the Lysholm score was 84; regarding the IKDC scoring scale, the patient was placed in group B.

**Fig. 4:**
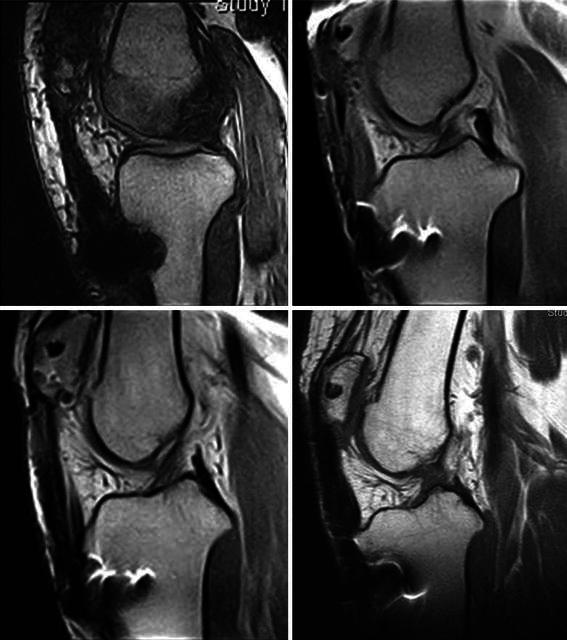
MRI images at 3, 6, 12, and 24 months postop, showing the evolution of the signal of the reconstructed patellar tendon

Radiograph images showed a satisfactory height of the patellar bone; the MRIs showed distinct progressive homogeneous improvement of the reconstructed tendon, and no signs of inflammation were detected (Fig. 4).

The patient gave informed consent prior to being included in the study.

The study was authorized by the local ethical committee and was performed in accordance with the ethical standards of the 1964 Declaration of Helsinki, as revised in 2000.

## Discussion

An accurate and immediate diagnosis is essential for the effective treatment of bone patellar tendon ruptures. Regardless of the many surgical procedures that have been described [4, 5], all of the studies published thus far indicate that the sooner the surgical treatment is implemented, the better the final outcome [6]. Moreover, correct planning of the postoperative rehabilitative protocol is crucial for a successful outcome. While some authors suggest [7, 8] a slow rehabilitative protocol to minimize the risk of re-rupture, other authors [6, 9] state that an accelerated postop program does not affect the re-rupture rate incidence, and lowers the risk of losing range of motion.

The case report described here shows how the appropriate surgical treatment and the subsequent rehabilitation program are key to a successful outcome, especially in the case of a chronic injury. Critically reviewing the history of our patient, many doubts about the first surgical procedure performed arise: as the correct diagnosis for this patient was missed at the beginning, a simple end-to-end suture of the tendon must have been considered a risky choice, associated with a high chance of recurrence. Indeed, despite the slow postoperative rehabilitation performed, the tenorrhaphy failed. Then a second failure occurred, which could have been due to the poor quality of the residual tendon on which the bone-patellar-tendon graft was sutured. As shown by our preoperative MRI exam, the re-rupture of the revised tendon occurred in exactly the same place (in the midsubstance of the proximal third of the patellar tendon), while the bone stock of the allograft was in place and intact. Even in this case, a cautious rehabilitative protocol did not protect the surgical procedure. For these reasons, we chose a reconstruction involving the use of an augmentation provided by the gracilis and semitendinosus tendons in a figure-of-eight shape. Moreover, to provide better quality augmentation, we left their tibial insertion intact in order to get a better vascularized autograft and preserve a safe and strong distal insertion. Despite the satisfactory stability of the reconstruction performed, and despite appearance of the postoperative radiograph, which showed a good level for the patellar bone height, we decided on a slow postoperative protocol: the risk of a new tendon rupture was thought to be much more significant, and therefore we risked the loss of a few degrees in the range of motion. Respecting the biological time of repair required for the autograft was the first goal of our rehabilitative procedure.

Complete ruptures of the patellar tendon always represent a challenge for the surgeon, especially in patients professionally involved in sports activities, who would like an adequate and fast return to their preoperative activity level. A correct and prompt diagnosis, adequate planning of the operation, and then a suitable related rehabilitative protocol represent the key factors in a positive result.
